# Fast and simple procedure for fractionation of zinc in soil using an ultrasound probe and FAAS detection. Validation of the analytical method and evaluation of the uncertainty budget

**DOI:** 10.1007/s10661-015-5020-6

**Published:** 2015-12-14

**Authors:** Barbara Leśniewska, Katarzyna Kisielewska, Józefa Wiater, Beata Godlewska-Żyłkiewicz

**Affiliations:** University of Bialystok, Institute of Chemistry, Ciołkowskiego 1K, 15-245 Bialystok, Poland; Bialystok University of Technology, Faculty of Civil and Environmental Engineering, Wiejska 45A, 15-351 Bialystok, Poland

**Keywords:** Sequential extraction, Modified BCR procedure, Zinc fractions, Ultrasound probe, Uncertainty budged

## Abstract

A new fast method for determination of mobile zinc fractions in soil is proposed in this work. The three-stage modified BCR procedure used for fractionation of zinc in soil was accelerated by using ultrasounds. The working parameters of an ultrasound probe, a power and a time of sonication, were optimized in order to acquire the content of analyte in soil extracts obtained by ultrasound-assisted sequential extraction (USE) consistent with that obtained by conventional modified Community Bureau of Reference (BCR) procedure. The content of zinc in extracts was determined by flame atomic absorption spectrometry. The developed USE procedure allowed for shortening the total extraction time from 48 h to 27 min in comparison to conventional modified BCR procedure. The method was fully validated, and the uncertainty budget was evaluated. The trueness and reproducibility of the developed method was confirmed by analysis of certified reference material of lake sediment BCR-701. The applicability of the procedure for fast, low costs and reliable determination of mobile zinc fraction in soil, which may be useful for assessing of anthropogenic impacts on natural resources and environmental monitoring purposes, was proved by analysis of different types of soil collected from Podlaskie Province (Poland).

**Electronic supplementary material:**

The online version of this article (doi:10.1007/s10661-015-5020-6) contains supplementary material, which is available to authorized users.

## Introduction

The presence of zinc in agricultural soils is of increasing concern due to its health risks to plants, humans and animals as well as its adverse effects to soil ecosystems. Zinc occurs in soil at a very wide concentration range of 5–770 mg kg^−1^, with average values in the range of 60–89 mg kg^−1^ on dependence of type of soil (Kabata-Pendias 2011). The warning and critical limits for zinc in soil were set at 150 and 300 mg kg^−1^ (Council Directive 86/278/EEC 1986). High concentration of zinc in contaminated soil may cause serious phytotoxicity to plants and/or its entrance into food chain (Prasad and Freitas 2003; Finžgar et al. 2007). As zinc is present in soil in several chemical forms, its total content does not give enough information about its bioavailability. In order to differentiate between mobile and non-mobile metal soil fraction, several single step (Krasnodębska-Ostręga et al. 2006; Garcia et al. 2014) and sequential extraction schemes have been proposed. Among them, the methods based on Tessier’s (Tessier et al. 1979). BCR (Ure et al. 1993, Davidson and Delevoye 2001) or modified BCR (Rauret et al. 2000, Rusnak et al. 2010) procedures are most often used. Tessier’s method partitions metals into five sequential fractions of increasing order of solubility. BCR method, evaluated by the Community Bureau of Reference, is a three-stage extraction, which is considered to be more operationally effective than that of Tessier’s. During evaluation of the method, some variability was found in step 2 of the extraction, which has been eliminated by the method modification. The revised protocol, which involved use of increased concentration of hydroxylammonium chloride (0.5 mol L^−1^) and lower pH (1.5), improves the reproducibility of extraction of the reducible fraction of the soil matrix (Mossop and Davidson 2003). Although, this procedure is commonly used for fractionation of metals in soil (Golia et al. 2007). it is extremely time consuming. The total time of sample pretreatment exceeds 50 h. In order to reduce the time of analysis, the long period of mechanical shaking is replaced by extraction assisted with microwaves (Alonso Castillo et al. 2011; Canepari et al. 2005) or ultrasounds (Davidson and Delevoye 2001; Rusnak et al. 2010; Canepari et al. 2005; Pérez-Cid et al. 1998; Pérez-Cid et al. 1999; Arain et al. 2008; Kazi et al. 2006).

Application of ultrasound energy to soil sample dispersed in an extraction solution results in fragmentation of soil particles, disintegration of soil aggregates and increase in the surface area available for reactions with the extraction reagent. These effects shorten the operation times necessary to extract soil fraction and enhance “green” aspects of such procedure in terms of time and energy requirements (Bendicho et al. 2012). A literature review (Table 1) shows that both ultrasound bath (Davidson and Delevoye 2001; Arain et al. 2008; Kazi et al. 2006; Relić et al. 2013; Péreza et al. 2008) and ultrasound probe (Davidson and Delevoye 2001; Pérez-Cid et al. 1999) have been applied in sequential extraction procedures as sources of ultrasonic energy (Catalado and Wildung 1978; Ross 1994; Prasad and Freitas 2003). The extraction conditions vary in power/frequency of ultrasounds and sonication time. In general, the use of an ultrasound probe additionally reduces the total time of the sequential extraction procedure by factor of 2 or 3. However, some authors claim that ultrasonic energy did not affect all the types of solid samples in precisely the same way as conventional shaking. The difficulty of developing a rapid version of sequential extraction of soil sample is related to the different fractionation patterns obtained by ultrasonic-assisted extraction in comparison to conventional ones, mainly for the matrix elements. Improper extraction of metal from one fraction of soil disturbs its partition among others, which can easily lead to wrong final results.

**Table 1:** Review of ultrasound-assisted extraction methods based on BCR procedures used for determination of Zn fractions in soil and sediments

Ultrasound source	Extraction conditions: sonication time and sonication power (recovery of Zn)			Sonication time (total time of procedure)	Sample	Ref.
	F I: Water-, acid-soluble, exchangeable	F II: Reducible	F III: Oxidizable			
Bath	A: 30 min, 42 kHz (80–120% for soil, 102% for BCR)	B: 30 min, 42 kHz (23–62% for soil, 35% for BCR)	C: step 1: 1 h, step 2: 1 h, step 3: 30 min, 42 kHz (23–62% for soil, 107% for BCR)	1 h 30 min (3 h 30 min)	Soil and sediment, BCR-701	Relić et al. 2013
Bath	A1: 30 min (98 %)	B1: 30 min (98 %)	C2: step 1: 1 h, step 2: 1 h, step 3: 30 min (97 %)	1 h 30 min (3 h 30 min)	Sewage sludge	Kazi et al. 2006
Bath	A: 3 h, 200 W (69 %)	B: 1 h, 200 W (100 % for soil; 61 % for BCR)	C: step 1: 1 h, step 2: 1 h, step 3: 1 h, 200 W (100 % for soil; 92 % for BCR)	5 h (7 h)	Soil, lake sediment BCR-601	Davidson and Delevoye 2001
Probe	A: 3 min, continuous output at 105 W (62 %)	B: 5 min, pulsed output at 75 W (70 %)	C: step 1: 1 h, step 2: 1 h, step 3: 1 min, continuous output at 105 W (60–70 %)	9 min (2 h 9 min)	Lake sediment BCR-601	Davidson and Delevoye 2001
Probe	A1: 7 min, 20 W (100%)	B1: 7 min, 20 W (97%)	C3: step 1: 2 min, step 2: 6 min, 15 W (98%)	22 min (1 h 22 min)	Sediment sewage	Pérez-Cid et al. 1998
Probe	A1: 12 min, amplitude 50%	B3: 9 min, amplitude 50%	C3: step 1: 9 min, step 2: 6 min, amplitude 50%	36 min (1 h 36 min)	Sediment	Péreza et al. 2008
Probe	A: 15–17 min, sonication power 90%, (72–100%)				Sediment, dust	Rusnak et al. 2010
Probe	A: 7 min. 15 W (84–95 % for soil, 98 % for BCR)	B2: 10 min, 15 W (82–93 % for soil, 97 % for BCR)	C1: step 1: 4 min, 15 W, step 2: 6 min, 15 W (87–102 % for soil, 120 % for BCR)	27 min (1 h 27 min)	Soil, BCR–701	This work

Extraction solutions for soil fractions: A: 40 mL of 0.11 mol L^−1^ CH_3_COOH; A1: 20 mL of 0.11 mol L^−1^ CH_3_COOH; B: 40 mL of 0.1 mol L^−1^ NH_2_OH HCl (pH = 2); B1: 20 mL of 0.1 mol L^−1^ NH_2_OH HCl (pH = 2); B2: 40 mL of 0.5 mol L^−1^ NH_2_OH HCl (pH = 1.5); B3: 20 mL of 0.5 mol L^−1^ NH_2_OH HCl (pH = 1.5); C: step 1: 10 mL of 30 % H_2_O_2_ (pH 2) heating at 85 °C for 1 h; step 2: 10 mL of 30 % H_2_O_2_ (pH 2) heating at 85 °C for 1 h; step 3: 50 mL of 1 mol L^−1^ CH_3_COONH_4_ (pH 2); C1: step 1: 10 mL of 30% H_2_O_2_ (pH 2) heating at 85 °C for 1 h; step 2: 50 mL of 1 mol L^−1^ CH_3_COONH_4_ (pH 2); C2: step 1: 5 mL of 30 % H_2_O_2_ (pH 2) heating at 85 °C for 1 h; step 2: 5 mL of 30 % H_2_O_2_ (pH 2) heating at 85 °C for 1 h; step 3: 25 mL of 1 mol L^−1^ CH_3_COONH_4_ (pH 2); C3: step 1: 5 mL of 30 % H_2_O_2_ (pH 2) heating at 85 °C for 1 h; step 2: 25 mL of 1 mol L^−1^ CH_3_COONH_4_ (pH 2)

The environmental risk from heavy metals in a soil can be properly evaluated only on the basis of consistent, reliable and accurate results of the metal content in soil fractions. For this reason, the procedure, for which suitability for the intended purpose was demonstrated, should be used. The validation of sequential extraction procedure comprises the evaluation of the number of validation parameters (e.g. accuracy, precision, specificity, detection and quantification limit, linearity of calibration graphs) and the estimation of uncertainty budget for metal in each fraction of soil. In spite of the numerous articles published in this domain, no fully validated method has yet been established. Therefore, in this work, the process of validation of the fast ultrasound-assisted sequential extraction (USE) procedure with the use of ultrasonic probe for the determination of zinc fractions in soil was described in detail, uncertainty of measurements was estimated using modelling approach and the uncertainty budget for determination of Zn in soil fractions was calculated. As the goal of our studies was to obtain performance similar to that of a well-established method, the conventional (with mechanical shaking) modified BCR sequential extraction procedure was used for comparison. A systematic study of various working conditions of ultrasound probe on the efficiency of zinc extraction from soil was executed then optimal conditions were chosen. The developed USE procedure was applied for determination of zinc fractions by flame atomic absorption spectrometry (FAAS) in samples of soil collected from Podlaskie Province (Poland).

## Materials and methods

### Instrumentation and measurement conditions

The content of zinc in soil fractions was determined by means of an atomic absorption spectrometry with a flame (air-acetylene) atomization (Solaar M6, Thermo Scientific, UK). A zinc hollow cathode lamp (LabHut, UK) was operated at 10 mA current. The integrated absorbance signal of zinc was measured at 213.9 nm with a slit width of 0.2 nm using deuterium background correction.

The external calibration technique based on reagent-matched standard solutions was used for zinc quantification by FAAS.

An ultrasound probe, VCX 130 model (Sonics and Materials, USA) (max. power 130 W, max. frequency 20 kHz) was used in pulsed mode (on/off, 15 s/15 s) in USE procedure. The system during sonication was cooled down with flowing tap water. A centrifuge, MPW 250 model (Med. Instruments, Poland), was used for the centrifugation of extracts of soil samples.

### Reagents and materials

Acetic acid (80 %), hydroxylammonium chloride, ammonium acetate and hydrogen peroxide (30 %) were pure for analysis (POCh, Poland). Nitric acid (65 %) and hydrochloric acid (30 %) were Suprapur (Merck, Germany). Standard solutions for calibration were prepared from stock solution of zinc (1000 μg mL^−1^; Fluka, Germany). All solutions were prepared by gravimetric dilution. Ultrapure water obtained from Milli-Q system (Millipore, USA) was used throughout the work. All plastic materials and glassware were thoroughly rinsed with Milli-Q water, soaked for a few days in 2 mol L^−1^ nitric acid and finally rinsed several times with Milli-Q water.

Certified reference material of lake sediment BCR-701 (IRMM, Belgium), with certified amounts of trace elements isolated by the BCR sequential extraction procedure, was used for evaluation of accuracy of the method.

The samples of soil that considerably varied in category and pH were collected from Podlaskie Province (Poland). The samples were homogenized, air-dried, crushed and sieved using a 1-mm sieve and stored in polyethylene vessels. For procedure development, three soil samples were used: sample L-very light soil, pH_KCl_ 4.3; sample M-medium soil, pH_KCl_ 7.1; sample H-heavy soil, pH_KCl_ 4.3. The pseudo-total content of zinc in soil was determined by FAAS technique after extraction of soil samples with aqua regia according to standard methods PN-ISO 11047 (2001) and PN-ISO 11466 (2002).

### Extraction procedure

The three-stage modified BCR procedure (Rauret et al. 2000) was used for fractionation of zinc in soil. For extraction of fraction I (FI), water- and acid-soluble and exchangeable, 40 mL of 0.11 mol L^−1^ CH_3_COOH was used. For extraction of fraction II (FII), reducible (metal bound to iron and manganese oxides), 40 mL of 0.5 mol L^−1^ NH_2_OH · HCl (pH 1.5) was used. The fraction III (FIII), oxidizable (metal bound to organic matter and sulphides), was released by oxidation of the organic matter using 30 % H_2_O_2_ and re-extraction of mineralization products with 50 mL of 1 mol L^−1^ CH_3_COONH_4_ (pH 2). Always, 1 g of soil sample was extracted. After each step, the suspension was centrifuged at 3000 rpm for 15 min and the supernatant was separated from the solid phase and stored at 4 °C until analysis. The remaining solid residue was washed with 20 mL of ultrapure water, and the washing was discarded after centrifugation. Optimized conditions for ultrasound-assisted extraction (power of ultrasound probe and time of sonication) are outlined in Table 2.

**Table 2:** Chemical reagents and conditions for the developed ultrasound-assisted extraction procedure using an ultrasound probe for 1 g of soil sample

Fraction	Reagents	Extraction conditions
F I: Water-, acid-soluble, exchangeable	40 mL of 0.11 mol L^−1^ CH_3_COOH	7 min at 15 W
F II: Reducible	40 mL of 0.5 mol L^−1^ NH_2_OH HCl (pH 1.5)	10 min at 15 W
F III: Oxidizable	Step 1: 10 mL of 30 % H_2_O_2_ (pH 2), step 2: 50 mL of 1 mol L^−1^ CH_3_COONH_4_ (pH 2)	Step 1: 4 min at 15 W, 1 h at 85 °C, step 2: 6 min at 15 W

### Methodology

During method optimization, the zinc content (expressed in mg kg^−1^ dry weight) obtained by USE procedure using an ultrasonic probe (*c*_USE_) was compared with that obtained by conventional procedure (*c*_conv_) for each soil sample. The recovery of zinc from fractions of soil obtained by USE procedure was calculated according to the following equation: *R* = (*c*_USE_/*c*_conv_) 100 %. Typically, three independent extractions were performed for each sample and the relative standard deviation (RSD%) was generally below 12 %.

The accuracy of the conventional BCR procedure was confirmed by analysis of CRM BCR 701. The content of Zn in FI, FII and FIII fractions of soil was 215.1 ± 3.6, 117.1 ± 4.1 and 48.5 ± 1.6 mg kg^−1^, respectively. The comparison with certified values has shown that bias of the method for fractions FI, FII and FIII was 4.9, 2.6 and 6.1 %, respectively. The bias of both, conventional and USE, procedures was calculated as a ratio of difference of between the measured and certified values to certified value.

## Results and discussion

A major limitation of the sequential extraction BCR procedure for trace element fractionation is a long sample processing time. In this work, we used an ultrasonic probe for speeding up the extraction of zinc fraction from soil. The parameters influencing the efficiency of zinc extraction by ultrasounds, such as a sonication time and a power of the ultrasound probe, were optimized for each step of the procedure individually.

### Optimization of ultrasound extraction conditions

**Fig. 1:**
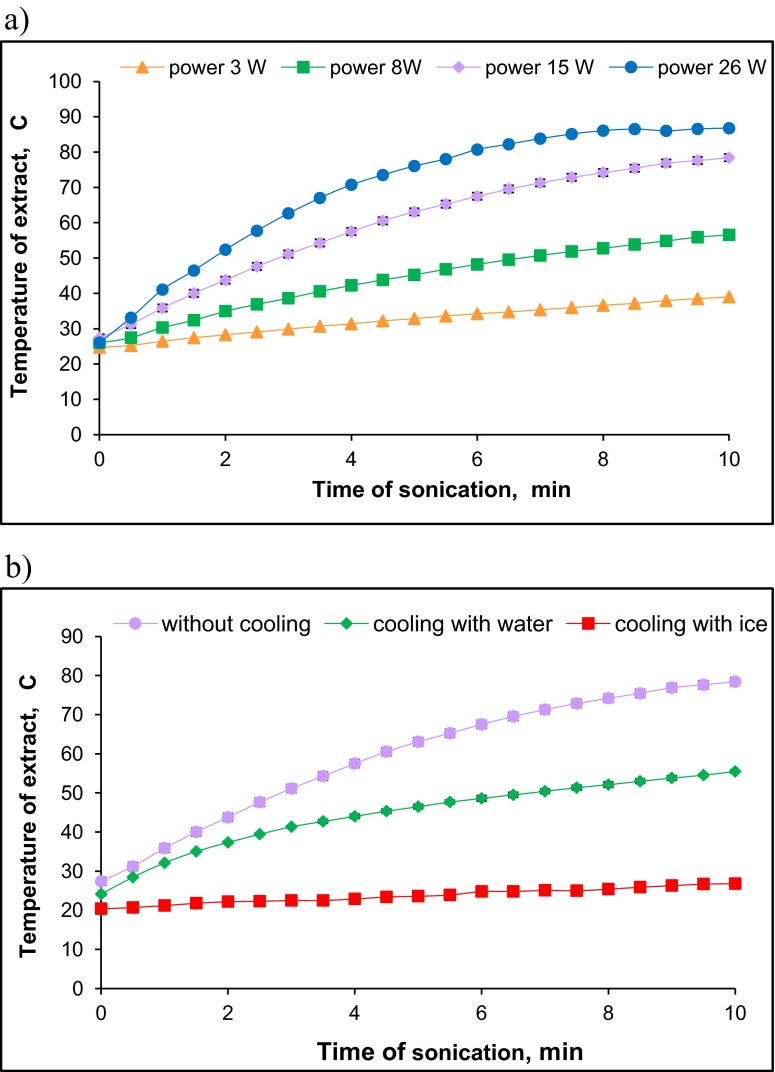
**a** Effect of the ultrasound probe power and sonication time on the temperature of soil extract, **b** effect of cooling mode (placing centrifuge tube in cold water or solid ice) on the temperature of soil extract within time (ultrasound probe power-15 W)

Initial experiments using an ultrasonic probe have shown that the temperature of the sample increases along with the increase of sonication time and ultrasound power (Fig. 1a). The temperature of extraction solution increased even to 80 °C within 6 min of sonication at 26 W. This phenomenon can considerably shift the liquid-solid phase equilibrium in the solution. The extraction of greater amounts of Cu, Ni, Pb and Zn was reported by Sahuquillo et al. (1999) at the temperature 40 than at 20 °C. Some authors reported that increase of temperature to 55 °C did not change the extractable amounts of Cd, Pb and Zn (Krasnodębska-Ostręga and Kowalska 2003). In order to keep constant temperature during sonication process, the cooling down of the centrifuge tubes with cold water (temp. 8–10 °C) or solid ice was tested. The temperature remained practically constant during sonication when solid ice was used, while placing the centrifuge tube in cold water allowed to keep the temperature below 50 °C (Fig. 1b). Because a frequent change of solid ice was laborious, a system with continuously flowing tap water (tubing wrapped around centrifuge tube) was constructed. Under such conditions, the temperature during extraction was kept below 25 °C.

**Fig. 2:**
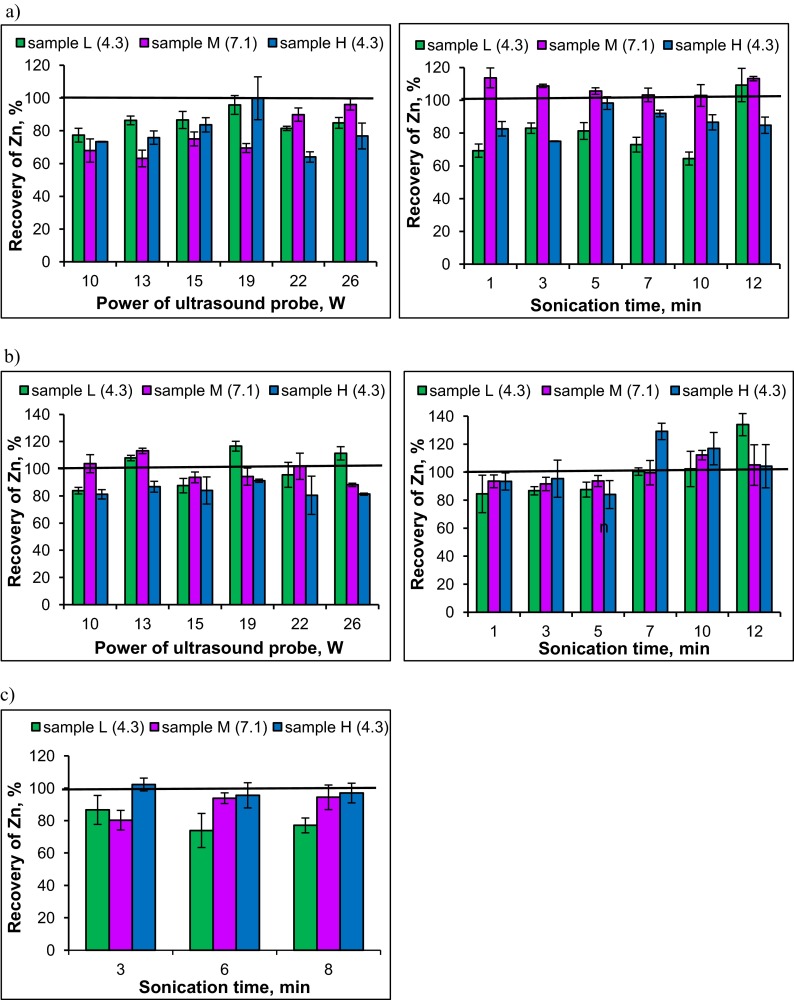
Recovery of Zn in fractions extracted from soil on dependence of the power of ultrasonic probe (fixed sonication time 5 min) and the sonication time (fixed sonication power 15 W): **a** fraction I, **b** fraction II, **c** fraction III (sample L-light soil, sample M-medium soil, sample H-heavy soil; in *brackets*, value of soil pH)

The influence of a power of an ultrasound probe on the recovery of zinc from fraction I was studied in the range 10 to 26 W at sonication time of 5 min. It was found that the recovery of analyte from tested samples was in the range 63–100 %, with the mean recovery for sample L equal to 85 %, for sample M equal to 77 % and for sample H equal to 79 % (Fig. 2a). The recoveries close to 100 % were obtained for power of 19 W, but taking into account the results obtained for determination of copper fractions in soil using USE (Leśniewska et al. 2014), as well as our efforts to develop the common extraction conditions for several metals, the ultrasound power of 15 W was chosen for further experiments. The influence of sonication time on the recovery of zinc from fraction I was studied in the range from 1 to 12 min. The recovery of analyte from acidic soils (samples L and H) was at the level 65–98 % for sonication time from 1 to 10 min. Quantitative recovery of analyte (103–108 %) from sample M (neutral pH) was achieved for 3–10 min of sonication time. In order to provide efficient extraction of zinc and to avoid its re-adsorption or liberation from the next fraction, the sonication was performed for 7 min (Fig. 2a).

The optimization of the extraction conditions of zinc from fraction II was performed analogously. The recovery of analyte from all samples was in the range 80 to 113 % for different powers of ultrasound probe tested (Fig. 2b). The mean recovery values for samples L and M were 100.5 ± 13.4 and 99.2 ± 8.9 %, respectively. Lower mean zinc recovery (84.2 ± 4.2%) was obtained for sample H. Also in this case, the ultrasound power of 15 W was chosen for further experiments. The recovery of analyte in the range of 84–134 % was obtained for all tested sonication times (Fig. 2b). Mean recovery values for samples L, M and H were 99.4 ± 18.8, 103.6 ± 10.8, and 110.0 ± 14.7 %, respectively. The sonication of samples for 10 min at 15 W was chosen as under these conditions acceptable recovery of zinc was obtained.

The optimization of the extraction conditions of zinc bound to fraction III was performed in two steps. First, the procedure of oxidation of the organic matter using 30 % H_2_O_2_ was studied. The conventional sample treatment (replicate heating of sample with H_2_O_2_ at 85 °C) was compared with the procedure applying only one addition of H_2_O_2_ and sonication of solution for 4 min (at 15 W) before its evaporation at 85 °C (Pérez-Cid et al. 1998). Next, the influence of sonication time (3, 6, 8 min) on the recovery of zinc during re-extraction of mineralization products with CH_3_COONH_4_ was tested (Fig. 2c). The lowest recovery of zinc (79.0 ± 6.6 %) was obtained for sample L, higher recoveries 89.5 ± 7.9 and 98.3 ± 3.5 % were obtained for samples M and H, respectively. The power of ultrasound probe chosen in this step was the same (15 W) as used before, while the sonication time was 6 min. The optimal parameters of extraction of zinc fractions of soil using an ultrasound probe are presented in Table 2.

The comparison of content of zinc fractions in soil samples determined using conventional and developed USE extraction method and FAAS detection is presented in Table 3. The developed procedure offers acceptable recovery of zinc fractions from tested soil samples of different properties (84–110 % from FI, 82–93 % from FII, 100–127 % from FIII). The repeatability of the results was in the range 1.9–13 %. The analysis of data presented in Table 1 shows that the developed procedure for the extraction of mobile fraction of zinc from soil using an ultrasound probe offers good recoveries of analyte and considerably shorter time (27 min sonication time, 87 min in total) than other proposed in the literature.

**Table 3:** Comparison of zinc fractions in soils obtained by conventional and ultrasound-assisted extraction methods

Soil sample (pH of soil)	Fraction	Content of Zn ± SD, mg kg^−1^, (*n* = 3)		Recovery ± SD, % (*n* = 3)
		Conventional BCR extraction	Ultrasound-assisted extraction	
Sample L (4.3)	F I	4.79 ± 0.33	4.53 ± 0.37	94.6 ± 7.6
	F II	2.33 ± 0.23	1.92 ± 0.19	82.5 ± 8.3
	F III	3.48 ± 0.29	3.48 ± 0.36	101.5 ± 10.5
Sample M (7.1)	F I	2.41 ± 0.24	2.28 ± 0.05	94.8 ± 1.9
	F II	2.34 ± 0.24	2.06 ± 0.04	87.9 ± 1.7
	F III	5.36 ± 0.14	4.67 ± 0.15	87.1 ± 2.8
Sample H (4.3)	F I	2.95 ± 0.16	2.48 ± 0.15	84.1 ± 5.2
	F II	2.42 ± 0.25	2.25 ± 0.30	93.1 ± 12.3
	F III	2.98 ± 0.20	2.98 ± 0.23	99.8 ± 7.9

Soil category are as follows: L-light, M-medium, H-heavy

### Validation of the USE procedure

Validation of the developed USE procedure was performed according to the international guideline ISO/IEC 17025:2005. The subsequent parameters were evaluated: linearity, limit of detection (LOD) and limit of quantification (LOQ), precision, trueness and uncertainty estimation. The assessment of uncertainty was carried out using a modelling approach. A full combined uncertainty calculation including possible sources of uncertainty was reported.

For the evaluation of the linearity of calibration graphs, the standards of Zn were prepared in the extraction solutions in the concentration range from 0.1 to 3 mg L^−1^. For all extraction solutions, the correlation coefficients of the calibration graphs were higher than 0.995 (Table 4) in the concentration range 0.1–1.6 mg L^−1^ and it was considered as a linear range of calibration graphs. The influence of used extraction solutions as well as the matrix of soil samples on the determination of zinc in soil fractions by FAAS was also examined. The results obtained by two calibration techniques: external calibration graph (based on zinc standards in extraction solution) and standard addition method (extracts of soil spiked with increasing amounts of zinc) were compared. The studies showed insignificant influence of soil components on analytical signals of zinc, as the slope of both calibration graphs were the same in the range of analytical error. Therefore, the external calibration procedure based on reagent-matched standard solutions was used for Zn quantification. In this way, the selectivity of the method was also confirmed.

**Table 4:** Validation parameters for ultrasound-assisted extraction method for determination of zinc fractions in soil

Validation parameter	Soil fraction: extraction solution		
	F I: 0.11 mol L^−1^ CH_3_COOH	F II: 0.5 mol L^−1^ NH_2_OH HCl	F III: 1 mol L^−1^ CH_3_COONH_4_
Linear range of calibration graph, mg L^−1^	0.028–1.6	0.040–1.6	0.025–1.6
Equation of calibration graph (R)	*y* = 0.1798*x* + 0.0034 (0.9996)	*y* = 0.1535*x* + 0.0054 (0.9993)	*y* = 0.1418*x* + 0.0034 (0.9982)
Limit of detection for extraction solution, mg L^−1^	0.011	0.020	0.014
Limit of quantification for extraction solution, mg L^−1^	0.028	0.040	0.025
Precision of absorbance measurements for soil extract as RSD, % (*n* = 6)	2.2	1.4	1.2
Limit of detection for soil fraction, mg kg^−1^	0.4	0.8	0.7
Limit of quantification for soil fraction, mg kg^−1^	1.1	1.6	1.2
Repeatability of Zn determination in BCR 701 fraction as RSD, % (*n* = 6)	4.8	2.7	4.9
Trueness of the procedure ^a^ Bias, % Found content ± *U*, *k* = 2, mg kg^−1^	−8.4 (−5.9^b^)	−0.6	20.6
	188 ± 16	113.3 ± 9.9	55.1 ± 6.4

^a^As compared to BCR 701: certified value ± *U*, *k* = 2, mg kg^−1^: F I 205.0 ± 6.0 (^b^192.9); F II 114.0 ± 5.0; F III 45.7 ± 4.0

^b^A mean value of 33 accepted data sets of Zn content in F I presented in [26]

The precision of measurements of zinc absorbance in extraction solutions, expressed as the relative standard deviation (RSD) for six independent measurements of the same sample was below 2.2 %. The repeatability of zinc determination in fractions I, II and III, expressed as RSD of six independent analyses of the same soil sample, was in the range from 2 to 13% (Table 3). This parameter is higher for the USE procedure than for the conventional one (2–10 %), what may be caused by a shorter extraction time resulting in different amounts of metal released from soil. The repeatability of zinc extraction from BCR 701 by USE method, expressed as RSD of results of analysis of six independent extraction of this sample, was in the range 2.7–4.9 %, whereas for conventional procedure was in the range 1.7–3.5 %. This parameter is better than that obtained for natural soil sample due to better homogeneity of the CRM. The limit of detection (LOD) was defined according to IUPAC recommendation as LOD = blank + 3SD_blank_, where SD is the standard deviation corresponding to six blank (extraction solution) measurements. For soil fractions, it was calculated using the volume of extraction solution and the mass of soil sample. The limit of quantification (LOQ) was calculated as LOQ = blank + 6SD_blank_.

For evaluation of trueness of the USE procedure, the content of zinc determined in fractions I, II and III of CRM BCR 701 (lake sediment) was compared with certified values. The bias of zinc content was in the range from −8.4 % (F I) to +20.5 % (F III). The similar value of the average bias of zinc content in F I, −5.9 %, was estimated on the basis of 33 literature data sets (mean value 192.9 mg kg^−1^) (Sutherland 2010), what indicated underestimation of zinc in this fraction in comparison to certified value. The greatest bias of zinc in F III is probably the result of incomplete extraction of analyte in earlier steps of procedure (F I and F II). The measurement of zinc content in this fraction was also problematic for several laboratories (Sutherland 2010). The differences in homogeneity of sample much strongly influence the efficiency of ultrasonic extraction than conventional modified BCR procedure, probably. The validation parameters of the method of determination of zinc in soil fractions by FAAS are summarized in Table 4.

The evaluation of measurement uncertainty of zinc fractionation in soil by developed USE procedure was performed according to the guide to the expression of uncertainty in measurement (GUM 2008) using modelling approach. This approach allows to identify and to estimate numerous possible components of uncertainty of the measurement procedure. After calculation of individual standard uncertainties of significant components of the analytical procedure, the combined standard uncertainty is estimated. The calculation of the uncertainty budget allows to indicate critical control points of the method.

The evaluation of expanded uncertainty of Zn content in F I is presented in detail in Electronic supplementary materials 1. In the same way, the expanded uncertainty of Zn content in F II and F III was evaluated. The content of zinc (*c*_Zn_ in mg/kg) in fraction I of soil extracted according to the developed USE procedure (outlined in Table 2) was defined as the measurand. A model equation used to calculate the result of the analysis (output quantity) on the basis of the measured parameters (input quantities) is as follows:

$$ {c}_{\mathrm{Zn}}=\frac{\left(\frac{A_s-a}{b}\right)\cdot {V}_e\cdot f}{m_s\cdot R} $$

where *c*_Zn_ denotes zinc content in F I of soil (mg kg^−1^); *A*_*s*_- the absorbance of Zn in F I extract solution; *a*- the intercept of the calibration curve, *b*- the slope of the calibration curve; *V*_*e*_- the volume of extract (L); *f- *the dilution factor of samples; *m*_*s*_- the mass of soil (g); *R- *recovery of Zn in fraction I of BCR 701.

**Fig. 3:**
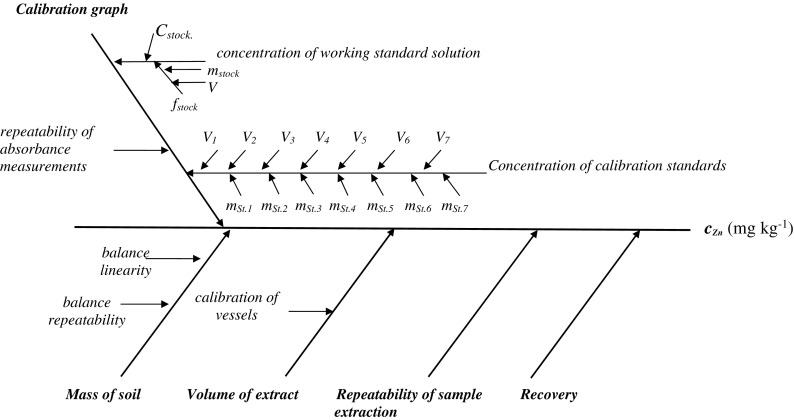
The Ishikawa diagram for Zn determination in fraction I (*c*_Zn_, mg kg^-1^) of BCR-701 by developed USE procedure. The symbols have the following meaning: *C*_stock_-concentration of Zn in stock solution, *m*_stock_-mass of stock solution, *f*_stock_-dilution factor for stock solution, *m*_st_, V-mass of standard solutions and final volume of standard solution

The sources of uncertainty are presented at an Ishikawa diagram (cause and effect diagram) (Fig. 3). The components significantly contributing to the measurement result are represented by main branches, which reflect the parameters in the model equation. The combined standard uncertainty of zinc content in fraction I of soil *u*_*c*_*(c*_Zn_*)* was calculated using standard uncertainty of all components and the law of propagation of uncertainty according to the following equation:

$$ \frac{u_c\left({c}_{\mathrm{Zn}}\right)}{c_{\mathrm{Zn}}}=\sqrt{{\left(\frac{u\left({m}_s\right)}{m_s}\right)}^2+{\left(\frac{u\left({V}_e\right)}{V_e}\right)}^2+{\left(u\left(\mathrm{cal}\right)\right)}^2+{\left(\frac{u(R)}{R}\right)}^2+{\left(\frac{u(f)}{f}\right)}^2+u{\left(\mathrm{repeat}{.}_{\mathrm{extr}.}\right)}^2} $$

where *u*(*m*_*s*_), *u*(*V*_*e*_), *u*(cal), *u*(*R*), *u*(*f*) and *u*(repeat._extr._) denote standard uncertainty of mass of soil, volume of extract, calibration, recovery, dilution factor and repeatability of extraction process, respectively.

To obtain an expanded uncertainty (*U*) of the result of a measurement at the 95 % confidence level, the combined standard uncertainty of Zn content in fraction I was multiplied by the coverage factor *k* of 2. The obtained values of relative uncertainty of each component, combined uncertainty and expanded uncertainty of Zn content in fractions I, II and III are presented in Table 5.

**Table 5:** The combined standard uncertainty and the expanded uncertainty of Zn content in fractions I, II and III of BCR 701 determined by ultrasound-assisted extraction method

Uncertainty		Fraction I	Fraction II	Fraction III
Relative uncertainty (percent contribution in combined uncertainty, %)	*u*(*m*_*s*_)/*m*_*s*_; *u*(*f*)/*f*; *u*(*V*_*e*_)/*V*_*e*_	(<0.05%)	(<0.05%)	(<0.05%)
	*u*(cal)	0.0206 (23.8%)	0.0269 (45.7%)	0.0175 (9.0%)
	*u*(repeat_.extr._)	0.0196 (21.4%)	0.0110 (7.7%)	0.0200 (11.7%)
	*u*(R)/R	0.0313 (54.7%)	0.0270 (46.3%)	0.0521 (79.3%)
	*u*_*c*_(c_*Zn*_)/c_*Zn*_	0.0423	0.0397	0.0585
*u*_*c*_(*c*_Zn_) (mg kg^-1^)		7.94	4.50	3.22
*c*_Zn_ ± U (mg kg^-1^), *k* = 2		188 ± 16	113.3 ± 9.0	55.1 ± 6.4
U (%), *k* = 2		8.5	7.9	11.7

In order to assess the analytical procedure, the uncertainty budget was constructed. The percentage contribution of uncertainty of each component in an combined uncertainty was calculated (as e.g. [*u*(*R*)*/R*]^2^*/*[*u*_*c*_(*c*_Zn_)*/c*_Zn_]^2^). As can be seen from the obtained results (Table 5), the zinc content in all fractions is strongly influenced by the recovery (46–79 %), the calibration graph (9–46 %) and the repeatability of extraction step (8–22 %). The uncertainty of the mass of soil, the volume of extract and dilution factor was neglected in combined uncertainty of Zn content in all cases. The most critical point of the developed procedure was the recovery of Zn from certified reference material BCR 701. The estimation of the concentration of analyte in extract solution calculated from calibration graph was also the important source of uncertainty. The uncertainty budget of that interim result shows that the repeatability of the analyte absorbance measurements in extract solutions and the slope of calibration graph were the most critical points of that step. Therefore, measurements of absorbance of analyte and the recovery experiment will require the greatest caution in the future work.

### Application of the USE procedure

**Fig. 4:**
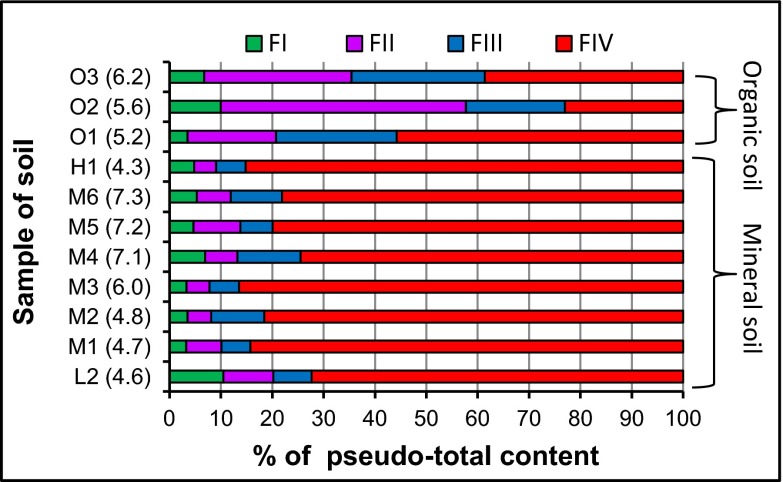
Partitioning of zinc in soil collected from Podlaskie Province after USE procedure (soil category: L-light, M-medium, H-heavy, O-organic; in *brackets*, the value of soil pH; F IV-residual fraction was calculated as the difference between pseudo-total content of Zn and content of Zn in fractions F I + F II + F III)

In order to test applicability of the developed USE method, 11 samples of soil of different properties collected from unpolluted arable land of Podlaskie Province (without external source of contamination) were analysed. The content of zinc fractions in soils as well as the pseudo-total content of zinc in soils after aqua regia digestion was determined by FAAS (Table 6). It was found that the pseudo-total content of zinc in all analysed samples did not exceed its permissible limit for agricultural soil in Poland (300 mg kg^−1^) (Ordinance of the Minister of Environment, Poland 2002) and was consistent with results obtained within monitoring programme for Podlasie Province (Zn content in the range 7.07–213 mg kg^−1^ for *n* = 241) (Report on the State of Environment of Podlasie Province, 2007). Hence, the analysed soil samples can be considered as unpolluted. The content of zinc in organic soils is higher than in mineral soils. Figure 4 shows the distribution of zinc into three fractions of soil obtained using developed USE method as well as in the residual, fraction IV. The content of zinc extracted by acetic acid represents only 3.3–10.5 % of the pseudo-total content in soil. Thus, the fraction of zinc bioavailable to plants is very low in all studied soils. The content of zinc in fraction II, associated with iron and manganese oxides, is also low and represents only 4.3–9.8 % of the pseudo-total content in mineral soils and 17–48 % in organic soils. The content of analyte in fraction III was slightly higher than in the first two fractions and represents 6–15 % of the pseudo-total metal content in mineral soil and 19–26 % in organic soil. The results confirmed zinc affinity to organic matter, in which it can be bound to functional groups of humic and fulvic acids. Metal associated with this fraction is assumed to remain in the soil for a long time; therefore, it is considered as not very mobile and available. The fraction IV (residual) made up the largest percentage in zinc total content, 54–87 % in mineral soil and 23–55 % in organic soil. The fractionation of zinc in collected soil samples should be considered as an example of natural distribution pattern of this metal. Similar distribution of zinc between the fractions of soil to that obtained in our work was reported in the literature (Száková et al. 1999; Moćko and Wacławek 2004; Zemberyova et al. 2006, Bakircioglu et al. 2011; Bielecka-Giełdoń et al. 2013). The decreasing order of the percentage share of zinc fraction in the pseudo-total content: F IV>F II, F III>F I was observed in soil collected from allotment garden in Koszalin (Bielecka-Giełdoń et al. 2013) and Opole (Poland) (Moćko and Wacławek 2004). A slightly different order of percentage share of analyte: F II>F IV>F III>F I and F II>F IV>F I>F III was observed in *Luvisols* collected from Siedlce Upland region (Pakuła 2011) and in *Endogleyic Phaeozem*, *Endogleyic Fluvisol and Haplic Fluvisol* Swiecka Plateau and Fordonska Valley (Poland) (Różański 2013), respectively, indicating higher binding of zinc with Fe and Mn oxides in such type of soil. The reports with other distribution patterns of zinc (F III>F II>F I>F IV or F III>F I>F II>F IV) were also published (Pérez-Cid et al. 1998, Davidson and Delevoye 2001; Kazi et al. 2006; Wiater et al. 2014). It can be concluded that the properties of soil samples such as soil category, content of clay and organic matter, pH of soil, grain-size composition and origin of zinc can significantly influence distribution of zinc in soil fractions. Therefore, the comparison of samples with different characteristics can lead to false conclusions.

**Table 6:** Content of zinc fractions in soil collected from Podlaskie Province after ultrasound assisted extraction

Sample	Soil category	*C*_org._, %	Soil pH	Content of Zn fractions in soil ± *U*, mg kg^−1^, *k* = 2			Pseudo-total content of Zn, mg kg^−1^
				F I	F II	F III	
L1	Light	1.3	4.6	2.61 ± 0.22	2.43 ± 0.18	1.84 ± 0.20	24.9
M1	Medium	1.5	4.7	1.22 ± 0.10	2.55 ± 0.19	2.12 ± 0.23	37.4
M2	Medium	1.6	4.8	1.19 ± 0.10	^a^1.55 ± 0.34	3.49 ± 0.38	33.8
M3	Medium	2.8	6.0	1.43 ± 0.12	1.92 ± 0.15	2.47 ± 0.27	43.0
M4	Medium	1.9	7.1	2.28 ± 0.19	2.06 ± 0.16	4.03 ± 0.44	32.8
M5	Medium	4.1	7.2	2.38 ± 0.20	4.66 ± 0.35	3.18 ± 0.35	50.9
M6	Medium	3.0	7.3	1.85 ± 0.16	2.30 ± 0.17	3.47 ± 0.38	34.8
H1	Heavy	4.3	4.3	2.48 ± 0.21	2.25 ± 0.17	2.98 ± 0.33	52.0
O1	Organic	24.8	5.2	1.69 ± 0.14	8.24 ± 0.63	11.27 ± 1.24	47.9
O2	Organic	36.6	5.6	6.86 ± 0.58	32.90 ± 2.50	13.31 ± 1.46	68.9
O3	Organic	27.3	6.2	4.66 ± 0.39	19.86 ± 1.51	17.99 ± 1.98	69.2

^a^Below LOQ of USE method (see Table 4), mean value ± standard deviation, *n* = 3

## Conclusions

In this work, the fast and simple USE method for determination of zinc fractions in soil was developed. This procedure was successfully used to assess the mobility of zinc in arable soil. The trueness of the developed USE method was confirmed by analysis of certified reference material BCR 701. The full validation of the method proved its usefulness for the determination of mobile zinc fractions in soil. A detailed evaluation of uncertainty budget indicated that recovery of zinc from CRM was the critical point of the developed measurement procedure. The determined content of zinc in soil fractions according to accelerated modified BCR procedure indicates the very low amount of its bioavailable fraction (F I).

The application of ultrasound probe for acceleration of extraction steps allows to reduce the total extraction time from 48 h to 27 min and the total operation time from 51 h to 87 min in comparison to conventional BCR procedure. The developed procedure significantly shortens operation time and energy consumption; therefore, it can be considered as a green method for fast assessing of the bioavailable and/or mobile fractions of zinc in soil, assessing of anthropogenic impact on soil or environmental monitoring purposes.

## Electronic supplementary material

ESM 1 (DOCX 34 kb)
