# Supplementary material for: Fast and simple procedure for fractionation of zinc in soil using an ultrasound probe and FAAS detection. Validation of the analytical method and evaluation of the uncertainty budget
Source: Environ Monit Assess. 2015 Dec 14;188:29. doi: 10.1007/s10661-015-5020-6 (PMC4679107; doi:10.1007/s10661-015-5020-6)
Supplement: Supplementary file 1 — (DOCX 34 kb) [file 10661_2015_5020_MOESM1_ESM.docx]

**Supplementary material**

Barbara Leśniewska^1^, Katarzyna Kisielewska^1^, Józefa Wiater^2^, Beata Godlewska-Żyłkiewicz^1^

Fast and simple procedure for fractionation of zinc in soil using ultrasound probe and FAAS detection. Validation of the analytical method and evaluation of the uncertainty budget

*^1^University of Bialystok, Institute of Chemistry, Ciołkowskiego 1K, 15-245 Bialystok, Poland*

*^2^Bialystok University of Technology, Faculty of Civil and Environmental Engineering, Wiejska 45A, 15-351 Bialystok, Poland*

*Measurement uncertainty and uncertainty budget*

The evaluation of measurement uncertainty of fractionation of zinc in soil by developed USE procedure was performed according to the Guide to the Expression of Uncertainty in Measurement (GUM) using modelling approach. This approach allows to identify and to estimate numerous possible components of uncertainty of the measurement procedure. After calculation of individual standard uncertainties of significant components of the analytical procedure, the combined standard uncertainty is estimated. The calculation of the uncertainty budget allows to indicate critical control points of the method. The evaluation of expanded uncertainty of Zn content in FI was presented herein in detail. In the same way the expanded uncertainty of Zn content in FII and FIII was evaluated. According to the GUM the evaluation of measurement uncertainty consists of seven steps. The content of zinc (*c_Zn_* in mg/kg) in fraction I of soil extracted according to the developed USE procedure was defined as the measurand. A model equation used to calculate the result of the analysis (output quantity) on the basis of the measured parameters (input quantities) is as follows:

$c_{Zn}=\frac{\left(\frac{A_{s}-a}{b}\right)\cdot V_{e}\cdot f}{m_{s}\cdot R}$ (1)

where: *c_Zn_* denotes zinc content in FI of soil (mg kg^-1^); *A_s_* – the absorbance of Zn in FI extract solution; *a* - the intercept of the calibration curve, *b* - the slope of the calibration curve; *V_e_* – the volume of extract (L); *f* - the dilution factor of samples; *m_s_* – the mass of soil (g); *R* – recovery of Zn in fraction I of BCR 701.

Next, value, unit, distribution, degree of freedom and the standard uncertainty were established for all input quantities. The set of input quantities was categorized to type A or B evaluation. Quantities whose values and uncertainties were directly determined in the current measurement were categorized to type A evaluation, whereas quantities whose values and uncertainties brought into the measurement from external sources as certificates of calibration standards, certified reference materials and used equipment (balance, pipettes, volumetric vessels) were categorized to type B evaluation.

The standard uncertainty related to the mass of soil samples was evaluated by using the values of linearity and repeatability of the balance from the calibration certificate (type B evaluation, rectangular distribution). The standard uncertainty of the volume of extract and standard solutions was evaluated by using the values taken from the producer’s certificate of pipettes and volumetric vessels (type B evaluation, rectangular distribution).

The concentration of zinc in extract of soil was calculated from the calibration graph. In the calibration step there are three sources of uncertainty: uncertainty of the preparation of calibration standard solutions *u(c_St._)*, the uncertainty of the repeatability of measurements of absorbance of standards *u(repeat_.A_)* and the uncertainty of linear regression graph *u(cal)* used for calculation of zinc concentration. The relative uncertainty of preparation of calibration standard solutions was determined using relative standard uncertainty of the concentration of zinc stock solution *u(c_Stock_)/c_Stock_* and its dilution factor *u(f_Stock_)/f_Stock_,* relative standard uncertainties of mass of working solutions used for preparation of calibration standards: *u(m_St1_)/m_St1_;* *u(m_St2_)/m_St2_*; *u(m_St3_)/m_St3_*; *u(m_St4_)/m_St4_*; *u(m_St5_)/m_St5_*; *u(m_St6_)/m_St6_*; *u(m_St7_)/m_St7,_* and their volumes *u(V)/V.* For the evaluation of the uncertainty of mass of working solutions *u(m_St._)* the data from calibration certificate of the balance (linearity and repeatability) were used. The adequate expression of *u(c_St._)/c_St._* is presented below:

 (2)

The standard uncertainty of repeatability of the absorbance measurement of calibration standards *u(repeat._A_)* was calculated as standard deviation of the mean absorbance *(s_A_*) measured in standard solutions for n=6 according to equation (3) .

$u(repeat._{A})=\frac{s_{A}}{\overline{A}\sqrt{n}}$ (3)

Standard uncertainty due to the regression graph was determined as:

 (4)

where: *s_xy_* is the residual standard deviation, *b* - the slope of the calibration curve, *p* - the number of measurements for a certain sample, *n -* the total number of standard solutions in the calibration curve, *A_sample_* - the mean absorbance of sample, $\bar{A}$- the mean absorbance of standard solutions, *c_i_* - the concentration of individual standard solution and $\bar{c}$ - the mean concentration of standard solutions.

The standard uncertainty of Zn recovery from BCR 701, *u(R),* was calculated using relative uncertainty of the measured value and relative uncertainty of the certified value. For calculation of the standard uncertainty of the measured value (type A evaluation) the standard deviation of the mean of observed results and number of measurements were used. For certified value, the half-width of confidence interval was used as a standard uncertainty.

$\frac{u(R)}{R}=\sqrt{\left(\frac{u(c_{measur.})}{c_{measur.}}\right)^{2}+\left(\frac{u(c_{ref.})}{c_{ref.}}\right)^{2}}$ (5)

The uncertainty due to repeatability of extraction step *u(repeat_.extr_.)* was taken into account of combined uncertainty and expressed as standard deviation of the mean of zinc content in fraction I of certified reference material BCR 701.

For evaluation of the uncertainty of dilution factor *u(f)* of BCR 701 extract the standard uncertainty of the pipette and the volumetric flask were used.

In the fifth step the values of standard uncertainty of all components were used for calculation of combined standard uncertainty of zinc content in fraction I of soil *u_c_(c_Zn_)* using the propagation principles according to the following equation:

$\frac{u_{c}(c_{Zn})}{c_{Zn}}=\sqrt{\left(\frac{u(m_{s})}{m_{s}}\right)^{2}+\left(\frac{u(m_{e})}{m_{e}}\right)^{2}+\left(u(cal)\right)^{2}+\left(\frac{u(R)}{R}\right)^{2}+\left(\frac{u(f)}{f}\right)^{2}+u(repeat._{extr.})^{2}}$ (6)

In the sixth step, to obtain an expanded uncertainty *(U)* of the result of a measurement at the 95 % confidence level, the combined standard uncertainty of Zn content in fraction I was multiplied by the coverage factor *k* of 2. The obtained values of relative uncertainty of each component, combined uncertainty and expanded uncertainty of Zn content in fraction I, II and III are presented in Table 5. In the seventh step the uncertainty bugged was constructed. The percentage contribution of uncertainty of each component in an combined uncertainty was calculated (as e.g. *[u(R)/R]^2^/[u_c_(c_Zn_)/c_Zn_]^2^*). The critical points of procedure were indicated as it was described in the text of article.
